# Implante Percutâneo de Balão Intra-Aórtico por Via Axilar em Pacientes Hospitalizados por Insuficiência Cardíaca: Experiência Inicial Brasileira

**DOI:** 10.36660/abc.20250735

**Published:** 2026-09-25

**Authors:** Bruno Alves da Mota Rocha, Pedro H. M. C. de Melo, Luis Mapa, Rafael Nunes Oliveira, Antônio Fernando Freire, Hernán Patricio García Mejía, Vanessa Corcioli Bellini, Liliane Kopel, Silvia Gelás Lage, Fabiana Goulart Marcondes-Braga, Sandrigo Mangini, Monica S. Ávila, Iascara Wozniak de Campos, Fernando Bacal, Fábio Sândoli de Brito, Alexandre A. Abizaid, Pedro Felipe Gomes Nicz

**Affiliations:** 1 Hospital das Clínicas Faculdade de Medicina Universidade de São Paulo São Paulo SP Brasil Instituto do Coração do Hospital das Clínicas da Faculdade de Medicina da Universidade de São Paulo, São Paulo, SP – Brasil; 2 Universidade Federal do Paraná Curitiba PR Brasil Universidade Federal do Paraná, Curitiba, PR – Brasil

**Keywords:** Insuficiência Cardíaca, Balão Intra-Aórtico, Coração Auxiliar, Transplante de Coração

## Abstract

**Fundamento:**

O balão intra-aórtico (BIA) é o dispositivo de assistência circulatória mecânica mais amplamente utilizado em pacientes com insuficiência cardíaca (IC) avançada em nosso meio; entretanto, o implante por via femoral limita a mobilidade do paciente durante o suporte.

**Objetivo:**

Relatar a experiência inicial de um centro de referência em IC avançada no Brasil com o implante percutâneo de BIA por via axilar.

**Métodos:**

Foram incluídos pacientes consecutivos hospitalizados por IC e submetidos ao implante percutâneo de BIA por via axilar em um centro de referência em cardiologia. Valores de p < 0,05 foram considerados estatisticamente significativos.

**Resultados:**

Entre dezembro de 2020 e dezembro de 2023, 22 pacientes foram submetidos ao implante percutâneo de BIA por via axilar. A média de idade foi de 53 ± 10,7 anos. Todos os pacientes encontravam-se hospitalizados por IC refratária e em lista de espera para transplante cardíaco. O implante foi realizado com sucesso em todos os pacientes com indicação da via axilar. A mediana do tempo de suporte foi de 15 dias (intervalo interquartil: 7-28 dias). As complicações observadas incluíram deslocamento do dispositivo (18,2%), isquemia de membro (9,1%) e eventos infecciosos e neurológicos isolados. Metade dos pacientes evoluiu para transplante cardíaco ou desmame do dispositivo.

**Conclusão:**

A experiência inicial com o implante percutâneo de BIA por via axilar demonstrou que a técnica é viável, segura e reprodutível. Estudos adicionais são necessários para definir o papel dessa estratégia na linha de cuidado de pacientes hospitalizados por IC avançada em lista de espera para transplante cardíaco.

## Graphical abstract

**Figura Central:**
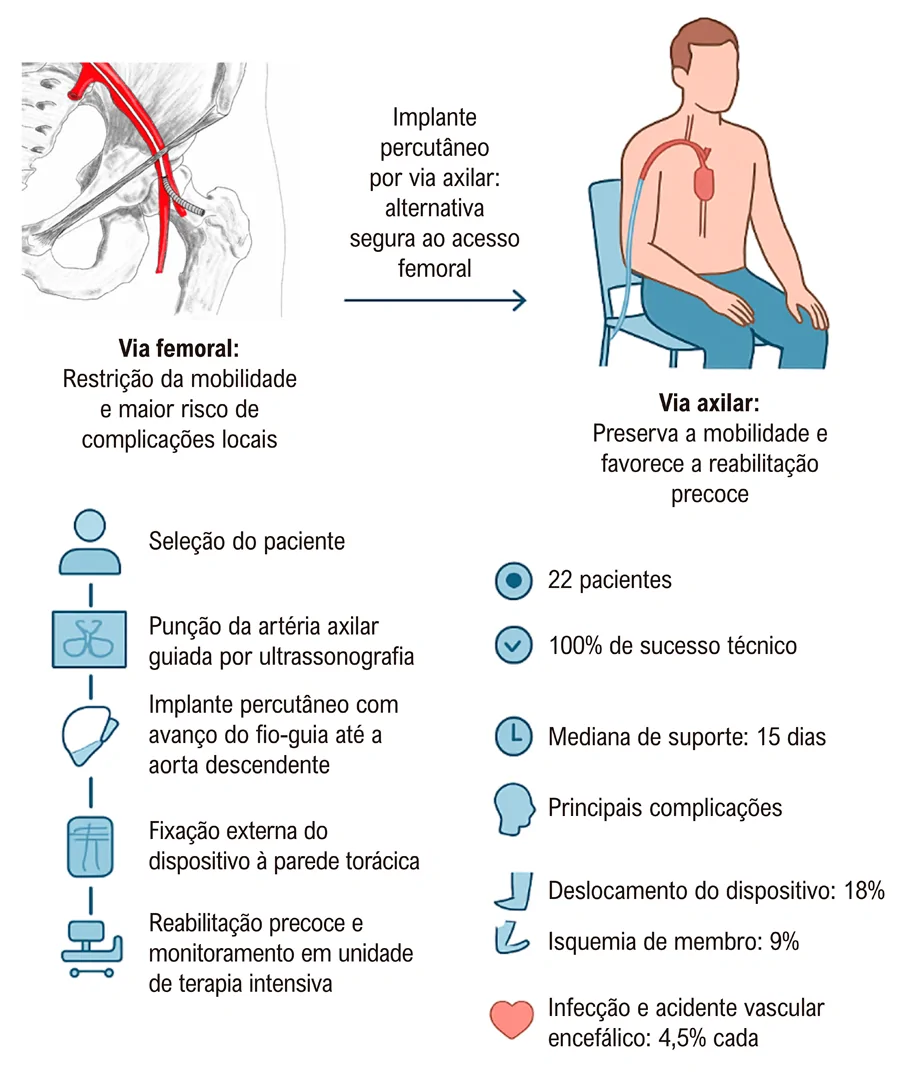
: Implante Percutâneo de Balão Intra-Aórtico por Via Axilar em Pacientes Hospitalizados por Insuficiência Cardíaca: Experiência Inicial Brasileira

O implante percutâneo de balão intra-aórtico por via axilar representa uma alternativa ao acesso femoral, permitindo a preservação da mobilidade e a realização de reabilitação precoce durante o suporte circulatório. Nesta série de 22 pacientes hospitalizados por insuficiência cardíaca avançada, o procedimento apresentou taxa de sucesso técnico de 100%, mediana de suporte de 15 dias e baixa incidência de complicações, demonstrando a viabilidade e a segurança da técnica.

## Introdução

O uso de dispositivos de assistência ventricular (DAVs) em pacientes com insuficiência cardíaca (IC) avançada tem como objetivo otimizar os parâmetros hemodinâmicos por meio da melhora do débito cardíaco, podendo ser empregado como ponte para o transplante cardíaco, terapia de destino ou ponte para recuperação.^1^

Entre os diversos DAV disponíveis, o balão intra-aórtico (BIA) destaca-se como o DAV mais amplamente utilizado em razão do menor custo e da maior facilidade de implante em comparação com outras modalidades de assistência circulatória mecânica. A técnica convencional de implante do BIA, por via transfemoral percutânea, é amplamente utilizada e pode ser realizada à beira do leito, dispensando o transporte do paciente para a sala de hemodinâmica ou o centro cirúrgico. Após o implante por via transfemoral, recomenda-se que o paciente permaneça em decúbito dorsal, com restrição da mobilidade e imobilização do membro inferior. Entretanto, essa condição prolongada está associada a desfechos desfavoráveis, como redução da mobilidade e perda de massa muscular.^2,3^

Como alternativa ao acesso femoral, McBride et al., em 1989, descreveram a técnica de implante cirúrgico do BIA por via axilar esquerda, possibilitando a deambulação e a mobilização de pacientes com IC avançada.^4^ Posteriormente, essa técnica foi aperfeiçoada para uma abordagem exclusivamente percutânea, com resultados promissores quanto à sua factibilidade, segurança e aos potenciais benefícios clínicos.^5^

Sabe-se que o perfil demográfico dos pacientes com IC, bem como a disponibilidade de DAVs, varia entre os países em função de aspectos regulatórios e das características de cada sistema de saúde. Embora o implante percutâneo de BIA por via axilar já esteja incorporado à prática clínica em alguns centros especializados, as evidências sobre sua utilização ainda são limitadas. Além disso, apesar de essa técnica já ser empregada em alguns centros brasileiros de referência em IC, seus resultados no contexto nacional ainda não foram estabelecidos.^6^

Assim, o presente estudo teve como objetivo relatar a experiência inicial com o implante percutâneo de BIA por via axilar em um centro brasileiro de referência em IC avançada e transplante cardíaco.

## Métodos

Trata-se de um estudo ambispectivo (retrospectivo para os pacientes submetidos ao procedimento antes da aprovação pelo comitê de ética em pesquisa com seres humanos e prospectivo para aqueles incluídos após essa aprovação), que incluiu todos os pacientes submetidos ao implante de BIA por via axilar em um único centro de referência em IC avançada e transplante cardíaco. Os pacientes encontravam-se hospitalizados por IC descompensada, em avaliação e em lista de espera para transplante cardíaco, sendo a indicação do dispositivo e da via de acesso definida pela equipe assistente. Foram coletados dados demográficos, clínicos, de exames complementares e relacionados ao procedimento. O seguimento clínico e os cuidados pós-implante foram conduzidos de acordo com a rotina assistencial da instituição.

Atualmente, uma equipe multidisciplinar composta por cardiologistas, intensivistas, infectologistas, cardiologistas intervencionistas, cirurgiões cardíacos, fisioterapeutas, psicólogos, enfermeiros, nutricionistas e assistentes sociais atua como rede de suporte aos pacientes críticos em avaliação ou em lista de espera para transplante cardíaco. Com base em decisão consensual da equipe multidisciplinar, pacientes com imobilização prolongada, sarcopenia, trombose ou infecção no sítio de inserção femoral foram preferencialmente encaminhados para o implante de BIA por via axilar.

### Anatomia vascular

O posicionamento dos implantes por via axilar e femoral está ilustrado na Figura 1. A artéria axilar corresponde à continuação da artéria subclávia após a margem lateral da primeira costela e estende-se até a margem inferior do músculo redondo maior (Figura 2A). A artéria subclávia esquerda apresenta trajeto intratorácico, origina diversos ramos responsáveis pela irrigação do território toracovertebral e mantém íntima relação anatômica com o plexo braquial. Em contraste, a artéria axilar apresenta trajeto extratorácico, localizado posteriormente ao músculo peitoral menor, caracterizando-se como um segmento relativamente livre de ramificações vasculares de grande importância.^7^

**Figura 1:**
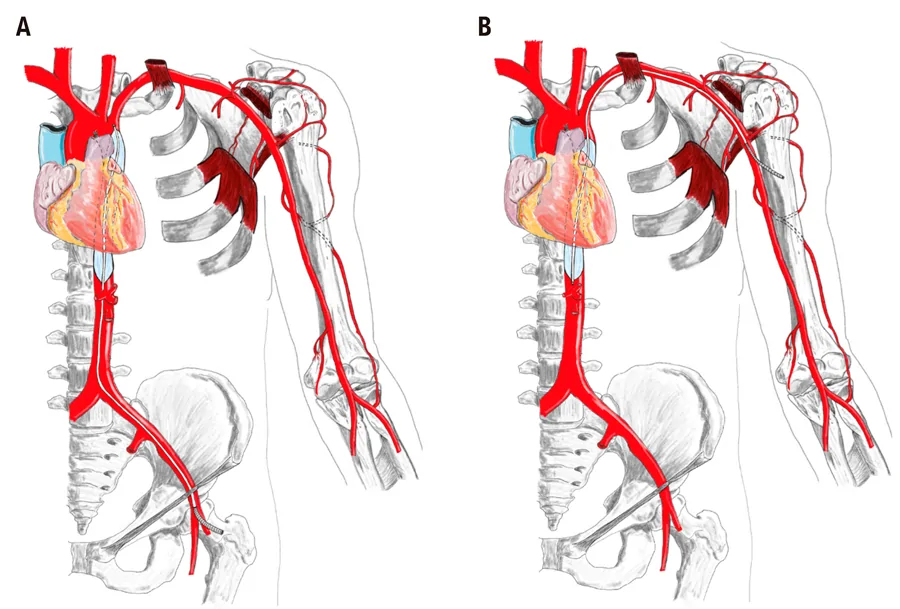
– Representação esquemática do posicionamento do balão intra-aórtico por via femoral (A) e por via axilar (B). Fonte: Elaboração própria.

**Figura 2:**
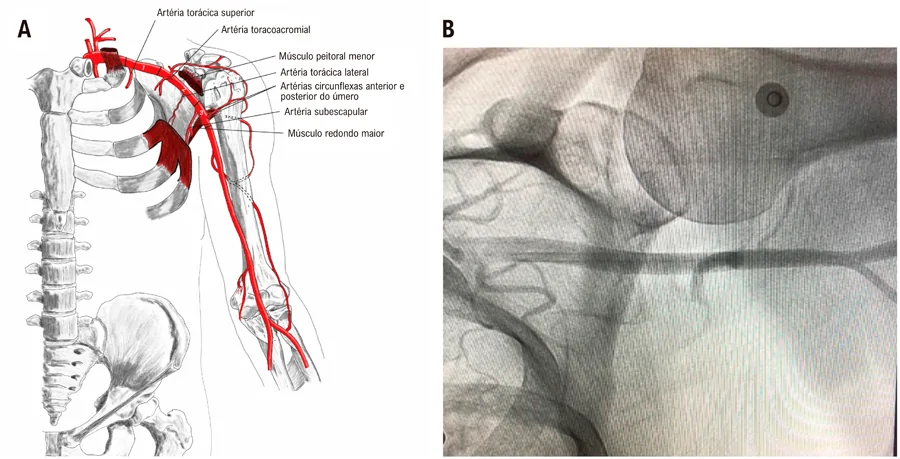
– A) Representação esquemática da anatomia da artéria axilar e de seus principais ramos. B) Angiografia da artéria axilar utilizada para definição do sítio ideal de punção para o implante do balão intra-aórtico, ao nível da cabeça do úmero, em segmento livre de ramificações arteriais. Fonte: Adaptado com permissão da referência Boros et al.6

Didaticamente, a artéria axilar é dividida em três segmentos pelo músculo peitoral menor^8^: i) primeiro segmento: localizado entre a margem lateral da primeira costela e a margem medial do músculo peitoral menor, encontra-se contido na bainha axilar e origina a artéria torácica superior; ii) segundo segmento: situa-se posteriormente ao músculo peitoral menor e origina dois ramos, as artérias toracoacromial e torácica lateral, que passam medial e lateralmente ao músculo, respectivamente; iii) terceiro segmento: estende-se da margem lateral do músculo peitoral menor até a margem inferior do músculo redondo maior e origina três ramos: a artéria subescapular (o maior ramo da artéria axilar) e as artérias circunflexas anterior e posterior do úmero.

Por razões ainda não completamente esclarecidas, a artéria axilar é acometida por doença aterosclerótica com frequência muito inferior à observada em outros territórios vasculares.^9^ Em conjunto, essas características anatômicas reduzem o risco de complicações vasculares e relacionadas ao acesso, como pneumotórax e hemotórax.

### Técnica de inserção axilar do balão intra-aórtico

Após a indicação do procedimento pela equipe multidisciplinar, os pacientes foram avaliados pelo cardiologista intervencionista responsável quanto à anatomia da região axilar e ao planejamento dos sítios de acesso. Todos os procedimentos foram realizados em sala de hemodinâmica, seguindo preparo semelhante ao de um cateterismo cardíaco diagnóstico. Preferencialmente, utilizou-se a artéria axilar esquerda, após avaliação ultrassonográfica que confirmasse sua perviedade e a ausência de placas ateroscleróticas ou de variações anatômicas na região de acesso. Para a punção, foi utilizado um suporte na altura do ombro para manter o membro superior em abdução. Realizou-se antissepsia da região axilar selecionada, bem como das regiões inguinais bilateralmente. Nos pacientes que já utilizavam BIA por via femoral, o sítio de inserção foi isolado, e o dispositivo somente foi removido após o posicionamento do BIA por via axilar.

As intervenções foram realizadas conforme a técnica descrita por Bhimaraj et al.,^10^ com algumas modificações. O acesso vascular foi obtido exclusivamente sob orientação ultrassonográfica, sem posicionamento prévio de fio-guia na artéria antes da punção axilar, evitando a necessidade de um acesso vascular adicional, como pela artéria radial.

A punção da artéria axilar foi realizada com agulha para acesso radial (introdutor 5F), guiada por ultrassonografia vascular. Em seguida, utilizou-se fluoroscopia para confirmar o posicionamento do fio-guia e do dilatador do introdutor radial inserido na artéria, permitindo a realização da angiografia (Figura 2B). Após confirmação do calibre adequado do vaso e da ausência de alterações anatômicas, posicionou-se o introdutor 5F na artéria e avançou-se o fio-guia do kit do BIA até a aorta descendente. Após o posicionamento distal do fio-guia, optava-se por uma de duas estratégias, conforme o calibre do vaso: implante do BIA com o introdutor específico de 7,5 Fr ou implante sem introdutor (*sheathless*). Em seguida, o introdutor 5F era removido e procedia-se ao implante do BIA nº 34 ou nº 40 (Maquet Datascope^®^), de acordo com a estratégia previamente definida. Não foi realizada antibioticoprofilaxia nem anticoagulação profilática com heparina.

A marca radiopaca proximal do BIA foi utilizada para determinar o posicionamento final do dispositivo, de forma semelhante à empregada no implante por via femoral. Nos pacientes que já se encontravam em suporte com BIA por via femoral, o dispositivo era colocado em modo de espera durante a manipulação da aorta descendente e somente removido após o adequado posicionamento do BIA por via axilar. A fixação externa do dispositivo foi realizada na parede torácica com pontos de sutura, evitando dobras no trajeto arterial e o consequente mau funcionamento do sistema.

### Cuidados pós-procedimento

Todos os pacientes em suporte com BIA permaneceram internados em unidade de terapia intensiva (UTI). O uso de antibióticos e de anticoagulação seguiu o protocolo institucional vigente para implante e troca de BIA por via transfemoral. Assim como nos implantes por via transfemoral, realizou-se radiografia de tórax à beira do leito imediatamente após o retorno do paciente à UTI.

Na ocorrência de alterações no padrão de funcionamento do dispositivo, novas radiografias de tórax foram realizadas para avaliação do posicionamento do BIA. Pequenos desvios de posicionamento foram corrigidos à beira do leito, enquanto ajustes mais complexos ou falhas de posicionamento foram avaliados em sala de hemodinâmica sob fluoroscopia.

Após um período de observação, os pacientes que apresentavam estabilidade hemodinâmica, ausência de infecção ativa e sem evidências de deterioração clínica recente eram estimulados a realizar atividades fora do leito, em posição ortostática, sob supervisão da equipe de fisioterapia (Figura 3).

**Figura 3:**
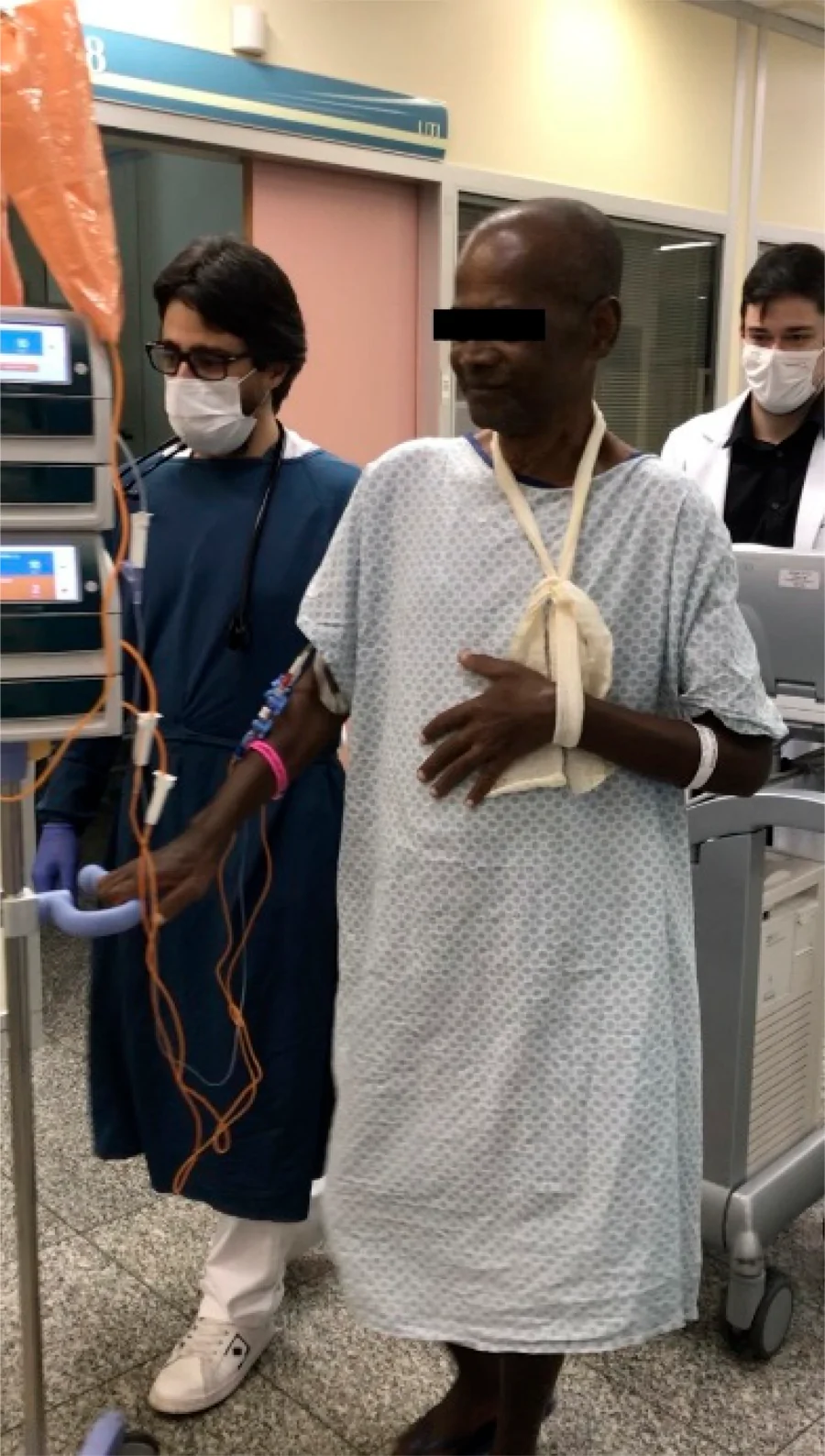
– Paciente em suporte com balão intra-aórtico por via axilar durante sessão de reabilitação cardiopulmonar sob supervisão da equipe assistencial. Fonte: Arquivo do Serviço de Hemodinâmica do Instituto do Coração do Hospital das Clínicas da Faculdade de Medicina da Universidade de São Paulo.

### Retirada do dispositivo

A retirada do dispositivo foi realizada no momento definido pela equipe assistente, de acordo com a evolução clínica de cada paciente. O procedimento seguiu a rotina institucional para remoção de introdutores femorais, evitando-se sua realização durante o efeito de medicamentos anticoagulantes ou na presença de níveis elevados de pressão arterial.

Após a interrupção da insuflação do BIA, o cateter foi removido em conjunto com o introdutor, quando utilizado, mantendo-se o membro superior em abdução. Hematomas ou sangramentos ativos no sítio de punção foram prontamente controlados por meio do ajuste do ponto de compressão.

### Análise estatística

Foi realizada análise descritiva dos dados. As variáveis categóricas foram apresentadas em frequências absolutas e relativas. As variáveis contínuas foram descritas por medidas de tendência central e de dispersão, conforme sua distribuição, avaliada pelo teste de normalidade de Shapiro-Wilk. As variáveis com distribuição normal foram expressas como média ± desvio padrão, enquanto aquelas com distribuição não normal foram apresentadas como mediana (intervalo interquartil [IIQ]).

As análises estatísticas foram realizadas no *software* RStudio para Windows (versão 2022.12.0+353.pro20, “Elsbeth Geranium”). Valores de p < 0,05 foram considerados estatisticamente significativos.

### Aspectos éticos

O estudo foi aprovado pelo comitê de ética em pesquisa com seres humanos sob CAAE 74190123.2.0000.0068, em conformidade com a Resolução nº 466/2012 do Conselho Nacional de Saúde, que regulamenta as pesquisas envolvendo seres humanos no Brasil. Todos os pacientes assinaram o termo de consentimento livre e esclarecido para a realização do procedimento. Os dados foram tratados de forma confidencial, em conformidade com a Lei Geral de Proteção de Dados.

## Resultados

Entre dezembro de 2020 e dezembro de 2023, foram incluídos 22 pacientes consecutivos submetidos ao implante de BIA por via axilar em um centro brasileiro de referência em IC avançada.

As características clínicas e demográficas da amostra estão apresentadas na Tabela 1. A média de idade foi de 53 ± 10,7 anos (variação: 27-75 anos), e 18 pacientes eram do sexo masculino. As etiologias mais frequentes da disfunção miocárdica foram miocardiopatia dilatada idiopática (45,5%), miocardiopatia chagásica (27,3%) e miocardiopatia isquêmica (13,6%). Todos os pacientes apresentavam IC em estágio avançado. Quase todos estavam em uso de dobutamina (21 pacientes), dos quais três recebiam terapia combinada com milrinona. Um total de 10 pacientes (45,5%) utilizavam vasodilatadores intravenosos ou orais, e cinco (22,7%) encontravam-se em uso de noradrenalina no momento do procedimento. A média da fração de ejeção do ventrículo esquerdo (FEVE) foi de 24,9% ± 7,6%.

**Tabela 1:** – Características basais da amostra

Variável	Resultado (n = 22)
Idade, anos	53 ± 10,7
Sexo masculino, n (%)	18 (81,8)
Classe funcional NYHA III ou IV, n (%)	22 (100)
Etiologia da IC, n (%)	
Miocardiopatia dilatada idiopática	10 (45,5)
Miocardiopatia chagásica	6 (27,2)
Miocardiopatia isquêmica	3 (13,6)
Outras	3 (13,6)
Uso de inotrópicos, n (%)	21(95,5)
IMC (kg/m^2^)	23,0 ± 3,9
FEVE (%)	24,9 ± 7,6
IM moderada a importante, n (%)	12 (54,5)
HAS, n (%)	11 (50,0)
DM, n (%)	7 (31,8)
*Clearance* de creatinina (ml/min)	60 (IIQ: 56-87)
DAOP, n (%)	2 (9,1)

DAOP: doença arterial obstrutiva periférica; DM: diabetes melito; FEVE: fração de ejeção do ventrículo esquerdo; HAS: hipertensão arterial sistêmica; IC: insuficiência cardíaca; IIQ: intervalo interquartil; IM: insuficiência mitral; IMC: índice de massa corporal; NYHA: New York Heart Association.

O implante foi realizado conforme planejado, por via axilar esquerda, em todos os casos, sem ocorrência de complicações intraprocedimento, correspondendo a uma taxa de sucesso técnico de 100%. O tempo médio total do procedimento foi de 62,8 ± 21,2 minutos. Os pacientes permaneceram em suporte com BIA por uma mediana de 15 dias (IIQ: 7-28 dias), com variação de 1-65 dias (Tabela 2). Durante o seguimento, 11 pacientes (50%) apresentaram evolução clínica favorável, definida por transplante cardíaco ou estabilização hemodinâmica com desmame progressivo do suporte circulatório (Figura Central).

**Tabela 2:** – Características do procedimento e evolução dos pacientes, em ordem consecutiva de inclusão

Paciente	Idade, anos	Número de BIAs prévios	Tempo de internação até o procedimento, dias	Duração do procedimento, min	Tempo de suporte com BIA por via axilar, dias	Complicações
1	58	1	45	74	9	Pseudoaneurisma da artéria axilar (1,5 × 1,0 cm)
2	58	1	58	48	13	Isquemia do MSE
3	59	0	86	67	26	Mau posicionamento com necessidade de troca do BIA
4	68	1	5	92	7	—
5	64	2	74	58	3	Mau posicionamento sem necessidade de troca do BIA
6	51	2	155	40	29	—
7	52	1	69	88	13	Infecção com necessidade de antibioticoterapia intravenosa
8	57	1	53	50	35	AVE
9	75	1	21	77	1	—
10	40	2	93	54	18	—
11	44	1	100	83	65	—
12	45	2	73	50	31	—
13	47	2	151	40	10	—
14	54	1	42	39	9	Mau posicionamento sem necessidade de troca do BIA
15	51	1	124	23	5	Mau posicionamento com necessidade de troca do BIA
16	27	1	79	64	7	—
17	56	1	115	54	55	—
18	55	0	102	49	19	—
19	47	1	177	68	17	—
20	52	1	137	60	38	Isquemia do MSE
21	67	1	66	108	17	—
22	39	1	71	95	7	—

AVE: acidente vascular encefálico; BIA: balão intra-aórtico; MSE: membro superior esquerdo.

Ao longo do período de assistência circulatória, foram registrados nove eventos adversos. Dois pacientes (9,1%) apresentaram sinais de isquemia do membro superior esquerdo (MSE), um (4,5%) evoluiu com bacteremia e necessidade de antibioticoterapia, um (4,5%) apresentou acidente vascular encefálico (AVE) durante a retirada do dispositivo, um (4,5%) desenvolveu pseudoaneurisma da artéria axilar e quatro (18,2%) apresentaram deslocamento do dispositivo (Tabela 3).

**Tabela 3:** – Eventos adversos observados durante o suporte com balão intra-aórtico por via axilar

Evento adverso	n (%)
Mau posicionamento/deslocamento do dispositivo	4 (18,2)
Isquemia do membro superior esquerdo	2 (9,1)
Bacteremia com necessidade de antibioticoterapia intravenosa	1 (4,5)
Acidente vascular encefálico	1 (4,5)
Pseudoaneurisma da artéria axilar	1 (4,5)

## Discussão

Os resultados deste estudo descrevem a experiência inicial com o implante percutâneo de BIA por via axilar em um centro brasileiro de referência em IC avançada. Os principais achados foram: i) a técnica de implante percutâneo por via axilar mostrou-se reprodutível, com elevada taxa de sucesso técnico; ii) o acesso transaxilar possibilitou a mobilização precoce dos pacientes; e iii) o procedimento apresentou perfil de segurança favorável, com baixa taxa de complicações, mesmo durante a fase inicial de implementação da técnica no serviço.

A primeira série de casos de implante percutâneo de BIA por via axilar, publicada em 2013, incluiu aproximadamente 50 pacientes encaminhados para avaliação de transplante cardíaco.^5^ Nessa coorte, a duração média do suporte foi de 18 dias, e 84% dos pacientes foram submetidos ao transplante cardíaco. As complicações que demandaram intervenção incluíram um episódio de sangramento maior e dois casos de isquemia do MSE com necessidade de tratamento cirúrgico. Ajustes de posicionamento foram necessários em 44% dos pacientes, enquanto 20% apresentaram mau funcionamento do dispositivo, exigindo sua substituição. Não foram registrados óbitos, AVE ou infecções relacionadas ao dispositivo.

Em nossa casuística, a taxa de deslocamento ou mau funcionamento do dispositivo foi de 18,2% (n = 4), valor inferior ao descrito na literatura (36%-44%).^5,6,10^ Uma possível explicação para esse achado é que os dispositivos atualmente disponíveis não foram desenvolvidos especificamente para o acesso axilar, o que dificulta sua adaptação à anatomia da artéria subclávia e do arco aórtico. Dispositivos projetados especificamente para implante por via axilar, como o NuPulse IVAS (NuPulseCV, North Carolina), vêm sendo utilizados com resultados promissores no suporte circulatório de longa duração, podendo reduzir a incidência de mau funcionamento e deslocamento.^11^

Apesar da ocorrência desses eventos, todos apresentaram evolução benigna e, na maioria dos casos, foram solucionados com pequenos ajustes de posicionamento realizados à beira do leito, sem comprometimento do funcionamento do dispositivo. Apenas dois pacientes apresentaram deslocamento do BIA em direção à artéria axilar, impedindo sua insuflação completa e tornando necessária a troca do sítio de implante do BIA sob fluoroscopia. Em ambos os casos, não houve complicações vasculares, e os pacientes evoluíram posteriormente para desmame do suporte circulatório.

Dois pacientes (9,1%) apresentaram isquemia do MSE, caracterizada por redução da amplitude dos pulsos radial e ulnar. Em um desses casos, a ultrassonografia Doppler evidenciou trombose parcial da artéria axilar esquerda. Em ambos os pacientes, optou-se pela retirada do dispositivo, anticoagulação plena e aquecimento passivo do membro, com recuperação da perfusão e resolução completa do quadro clínico. Nenhum caso necessitou de abordagem cirúrgica vascular.

A isquemia de membro constitui uma complicação bem descrita após o implante de BIA, tanto por via axilar quanto por via femoral, apresentando incidência semelhante entre ambas as vias de acesso. A maior série de pacientes submetidos ao implante de BIA por via axilar publicada até o momento relatou taxa de isquemia de membro de 5,3%.^10^ Da mesma forma, uma revisão que incluiu 20 estudos envolvendo o implante de BIA por via femoral demonstrou incidência média de isquemia de membro de 8,03% (± 7,40%).^12^

Frequentemente, a retirada do dispositivo e o aquecimento passivo do membro são suficientes para reverter essa complicação. Dados provenientes, predominantemente, de estudos com BIA implantado por via femoral demonstram que a necessidade de amputação do membro é rara, com incidência de 0,1%-3,5%.^12^ A utilização de introdutores de maior diâmetro, o sexo feminino, a não utilização da técnica *sheathless* e o maior tempo de permanência do dispositivo estão entre os principais fatores associados à ocorrência de isquemia de membro.^12-16^ Boudoulas et al. demonstraram que a incidência dessa complicação aumenta de forma expressiva quando o tempo de permanência do dispositivo ultrapassa 2 dias.^13^ Ressalta-se que, em nossa série, os dois casos de isquemia ocorreram em pacientes do sexo feminino, ambas com tempo prolongado de suporte (13 e 38 dias).

Em um paciente da presente série ocorreu AVE durante a retirada do dispositivo, identificado após o surgimento súbito de déficit motor desproporcional em dimídio direito. O paciente foi prontamente submetido à trombólise intravenosa, seguida de trombectomia mecânica, evoluindo com recuperação completa do déficit neurológico. Durante a mesma internação, foi submetido com sucesso ao transplante cardíaco e recebeu alta hospitalar sem déficits neurológicos residuais.

A literatura descreve que o implante de BIA por via axilar pode estar associado a lesões neurovasculares, particularmente envolvendo o plexo braquial, manifestadas por dor neuropática, parestesias ou déficits motores transitórios. Essas complicações são atribuídas à proximidade anatômica entre a artéria axilar e as estruturas nervosas adjacentes, bem como à possibilidade de compressão local, formação de hematomas ou mau posicionamento do dispositivo. Séries clínicas e revisões indicam que esses eventos são relativamente infrequentes, mas devem ser considerados durante o planejamento do acesso e a execução da técnica.^8^ Em nossa amostra, embora não tenha sido realizada avaliação neurológica sistemática, não houve relato clínico de dor neuropática ou déficits neurológicos compatíveis com lesão do plexo braquial durante o seguimento. Esse achado está em concordância com estudos que demonstram baixa incidência de complicações neurológicas quando o implante axilar é realizado após adequada seleção anatômica e com técnica criteriosa.^10^

Boudoulas et al. publicaram uma análise retrospectiva envolvendo 195 pacientes submetidos ao implante percutâneo de BIA por via axilar.^10^ Com tempo médio de suporte de 19 dias, observou-se sucesso terapêutico em 68,2% dos pacientes, resultado semelhante ao descrito para o implante de BIA por via transfemoral. Apenas 11% dos pacientes necessitaram da retirada do dispositivo em decorrência de complicações.

Em nossa casuística, 50% dos pacientes apresentaram evolução clínica favorável, definida por transplante cardíaco ou desmame do suporte circulatório. Esse resultado deve ser interpretado considerando-se a elevada gravidade clínica da população estudada, caracterizada por média de FEVE de 25% (± 7,6%), uso de inotrópicos em praticamente todos os pacientes no momento do implante, múltiplos procedimentos invasivos prévios e tempo prolongado de internação antes da realização do procedimento (mediana de 76,5 dias).

Por fim, a preservação da mobilidade nesses pacientes merece especial atenção. Em candidatos ao transplante de órgãos sólidos, pior capacidade funcional está associada ao aumento das complicações perioperatórias e à recuperação pós-operatória mais prolongada, com impacto negativo sobre os desfechos após o transplante.^17^ Além disso, a capacidade de deambulação em pacientes hospitalizados por IC tem sido associada à redução do tempo de internação e da necessidade de readmissões hospitalares.^18^ Dessa forma, estratégias que favoreçam a manutenção da mobilidade e da capacidade funcional de pacientes com IC avançada em lista de espera para transplante cardíaco devem ser incentivadas. A Figura 3 ilustra o processo de deambulação e reabilitação motora de um paciente em suporte com BIA em UTI.

Em conjunto, os resultados desta série de casos sugerem que o implante percutâneo de BIA por via axilar constitui uma alternativa viável para pacientes com IC avançada. Estratégias que favoreçam a preservação da mobilidade e da capacidade funcional, como a utilização da via axilar, podem representar um avanço relevante no manejo de pacientes em lista de espera para transplante cardíaco.

### Limitações do estudo

As principais limitações deste estudo incluem seu delineamento unicêntrico, o reduzido tamanho da amostra e a ausência de comparação direta com outras vias de acesso. Estudos multicêntricos envolvendo um maior número de pacientes e a participação de outros centros brasileiros com experiência no manejo da IC avançada poderão contribuir para definir a viabilidade da incorporação dessa técnica à prática clínica de rotina em nosso meio.

## Conclusões

O presente estudo descreve a experiência inicial com a implementação da técnica de implante percutâneo de BIA por via axilar em um centro brasileiro de referência em cardiologia. Os resultados clínicos observados foram semelhantes aos descritos na literatura internacional e corroboram a viabilidade, a eficácia e a segurança dessa abordagem. Entretanto, estudos adicionais são necessários para definir o papel dessa intervenção na linha de cuidado de pacientes hospitalizados por IC avançada em lista de espera para transplante cardíaco.
