# Supplementary material for: Implante Percutâneo de Balão Intra-Aórtico por Via Axilar em Pacientes Hospitalizados por Insuficiência Cardíaca: Experiência Inicial Brasileira
Source: Arq Bras Cardiol. 2026 Sep 25;123(8):e20250735. [Article in Portuguese] doi: 10.36660/abc.20250735 (PMC13671729; doi:10.36660/abc.20250735)
Supplement: Translation

# Translation: Percutaneous Axillary Intra-Aortic Balloon Pump Implantation in Patients Hospitalized with Heart Failure: Initial Brazilian Experience

**DOI:** 10.36660/abc.20250735i

**Authors:** Bruno Alves da Mota Rocha, Pedro H. M. C. de Melo, Luis Mapa, Rafael Nunes Oliveira, Antônio Fernando Freire, Hernán Patricio García Mejía, Vanessa Corcioli Bellini, Liliane Kopel, Silvia Gelás Lage, Fabiana Goulart Marcondes-Braga, Sandrigo Mangini, Monica S. Ávila, Iascara Wozniak de Campos, Fernando Bacal, Fábio Sândoli de Brito, Alexandre A. Abizaid, Pedro Felipe Gomes Nicz

**Affiliations:** 1 Brazil Instituto do Coração do Hospital das Clínicas da Faculdade de Medicina da Universidade de São Paulo, São Paulo, SP – Brazil; 2 Brazil Universidade Federal do Paraná, Curitiba, PR – Brazil

**Keywords:** Heart Failure, Intra-Aortic Balloon Pumping, Heart-Assist Devices, Heart Transplantation

## Abstract

**Background:**

The intra-aortic balloon pump (IABP) is the most widely used mechanical circulatory support device for patients with advanced heart failure (HF) in our setting. However, femoral implantation restricts patient mobility during support.

**Objective:**

To report the initial experience of a Brazilian advanced HF referral center with percutaneous axillary IABP implantation.

**Methods:**

Consecutive patients hospitalized for HF who underwent percutaneous axillary IABP implantation at a tertiary cardiology referral center were included. A p-value < 0.05 was considered statistically significant.

**Results:**

Between December 2020 and December 2023, 22 patients underwent percutaneous axillary IABP implantation. The mean age was 53 ± 10.7 years. All patients were hospitalized with refractory HF and were listed for heart transplantation. The procedure was successfully performed in all patients selected for the axillary approach. The median duration of support was 15 days (interquartile range: 7–28 days). Observed complications included device migration (18.2%), limb ischemia (9.1%), and isolated infectious and neurological events. Half of the patients either underwent heart transplantation or were successfully weaned from mechanical support.

**Conclusion:**

The initial experience with percutaneous axillary IABP implantation demonstrated that the technique is feasible, safe, and reproducible. Further studies are needed to establish the role of this strategy within the continuum of care for patients hospitalized with advanced HF while awaiting heart transplantation.

## Introduction

The use of ventricular assist devices (VADs) in patients with advanced heart failure (HF) aims to optimize hemodynamic parameters by improving cardiac output and may be used as a bridge to heart transplantation, destination therapy, or bridge to recovery.^1^

Among the various VADs available, the intra-aortic balloon pump (IABP) remains the most widely used mechanical circulatory support device because of its lower cost and greater ease of implantation compared with other forms of mechanical circulatory support. The conventional technique for IABP implantation, performed via a percutaneous transfemoral approach, is widely adopted and can be carried out at the bedside, eliminating the need to transport the patient to the cardiac catheterization laboratory or operating room. Following transfemoral implantation, patients are generally required to remain in the supine position, with restricted mobility and immobilization of the lower limb. However, prolonged immobilization has been associated with adverse outcomes, including reduced mobility and loss of skeletal muscle mass.^2,3^

As an alternative to femoral access, McBride et al. first described the surgical implantation of an IABP through the left axillary artery in 1989, enabling ambulation and mobilization of patients with advanced HF.^4^ Subsequently, the technique evolved into a fully percutaneous approach, with promising results regarding its feasibility, safety, and potential clinical benefits.^5^

It is well recognized that the demographic profile of patients with HF, as well as the availability of VADs, varies across countries according to regulatory policies and healthcare system characteristics. Although percutaneous axillary IABP implantation has been incorporated into clinical practice at some specialized centers, evidence supporting its use remains limited. Furthermore, despite its adoption in several Brazilian referral centers for advanced HF, outcomes associated with this technique have not yet been established in the national setting.^6^

Therefore, the current study aimed to report the initial experience with percutaneous axillary IABP implantation at a Brazilian referral center for advanced HF and heart transplantation.

## Methods

This was an ambispective study (retrospective for patients who underwent the procedure before approval by the institutional human research ethics committee and prospective for those enrolled thereafter), including all patients who underwent axillary IABP implantation at a single referral center for advanced HF and heart transplantation. Patients were hospitalized with decompensated HF and were either undergoing evaluation or listed for heart transplantation. The indication for mechanical circulatory support and the choice of vascular access were determined by the treating team. Demographic, clinical, procedural, and ancillary test data were collected. Clinical follow-up and post-implant care were conducted according to the institution’s standard clinical practice.

The institution currently relies on a multidisciplinary team comprising cardiologists, intensivists, infectious disease specialists, interventional cardiologists, cardiac surgeons, physical therapists, psychologists, nurses, dietitians, and social workers to provide comprehensive care for critically ill patients undergoing evaluation or awaiting heart transplantation. Based on multidisciplinary consensus, patients with prolonged immobilization, sarcopenia, thrombosis, or infection at the femoral insertion site were preferentially selected for axillary IABP implantation.

### Vascular anatomy

The positioning of axillary and femoral implants is illustrated in Figure 1. The axillary artery is the continuation of the subclavian artery beyond the lateral border of the first rib and extends to the inferior border of the teres major muscle (Figure 2A). The left subclavian artery follows an intrathoracic course, gives rise to several branches supplying the thoracovertebral region, and lies in close anatomical proximity to the brachial plexus. In contrast, the axillary artery follows an extrathoracic course posterior to the pectoralis minor muscle, representing a vascular segment that is relatively free of major arterial branches.^7^

**Figure 1:** – Schematic representation of intra-aortic balloon pump positioning through the femoral approach (A) and the axillary approach (B). Source: Prepared by the authors.

**Figure 2:** – A) Schematic representation of the anatomy of the axillary artery and its major branches. B) Angiographic image of the axillary artery used to identify the optimal puncture site for intra-aortic balloon pump implantation, at the level of the humeral head, within an arterial segment free of major branches. Source: Adapted with permission from Boros et al.6

For descriptive purposes, the axillary artery is divided into three segments by the pectoralis minor muscle^8^: i) the first segment extends from the lateral border of the first rib to the medial border of the pectoralis minor muscle, lies within the axillary sheath, and gives rise to the superior thoracic artery; ii) the second segment lies posterior to the pectoralis minor muscle and gives rise to two branches, the thoracoacromial and lateral thoracic arteries, which pass medial and lateral to the muscle, respectively; and iii) the third segment extends from the lateral border of the pectoralis minor muscle to the inferior border of the teres major muscle and gives rise to three branches: the subscapular artery (the largest branch of the axillary artery) and the anterior and posterior circumflex humeral arteries.

For reasons that remain incompletely understood, the axillary artery is affected by atherosclerotic disease far less frequently than other vascular territories.^9^ Collectively, these anatomical characteristics reduce the risk of vascular and access-related complications, such as pneumothorax and hemothorax.

### Axillary intra-aortic balloon pump implantation technique

Following multidisciplinary approval of the procedure, patients were evaluated by the attending interventional cardiologist to assess axillary anatomy and plan the vascular access sites. All procedures were performed in the cardiac catheterization laboratory using a preparation protocol similar to that for diagnostic cardiac catheterization. The left axillary artery was preferentially selected after duplex ultrasonography confirmed vessel patency and the absence of atherosclerotic plaques or anatomical variations at the access site. During arterial puncture, the ipsilateral upper limb was maintained in abduction using a shoulder support. Antisepsis was performed over the selected axillary region and both groins. In patients already receiving femoral IABP support, the femoral insertion site was isolated, and the device was removed only after successful positioning of the axillary IABP.

Procedures were performed according to the technique described by Bhimaraj et al.,^10^ with several modifications. Vascular access was obtained exclusively under ultrasound guidance, without prior placement of a guidewire in the artery before axillary puncture, thereby eliminating the need for an additional vascular access site, such as the radial artery.

The axillary artery was punctured using a radial access needle (5-Fr introducer) under ultrasound guidance. Fluoroscopy was then used to confirm the position of the guidewire and dilator of the radial introducer within the artery, allowing angiographic assessment (Figure 2B). After confirmation of adequate vessel caliber and the absence of anatomical abnormalities, the 5-Fr introducer was positioned within the artery, and the guidewire included in the IABP kit was advanced into the descending aorta. Once the guidewire had been advanced distally, one of two implantation strategies was selected according to vessel diameter: IABP implantation using the dedicated 7.5-Fr sheath or sheathless implantation. The 5-Fr introducer was then removed, and either a 34-mL or 40-mL IABP (Maquet Datascope^®^) was implanted according to the predefined strategy. Neither antibiotic prophylaxis nor prophylactic heparin anticoagulation was administered.

The proximal radiopaque marker of the IABP was used to determine the final position of the device, following the same principles applied during transfemoral implantation. In patients already supported with a femoral IABP, the device was placed in standby mode during manipulation of the descending aorta and removed only after satisfactory positioning of the axillary IABP had been achieved. The external portion of the device was secured to the chest wall with sutures to prevent kinking along the arterial course and the resulting device malfunction.

### Postprocedural care

All patients receiving IABP support remained in the intensive care unit (ICU). The use of antibiotics and anticoagulation followed the institutional protocol for transfemoral IABP implantation and exchange. As with transfemoral implantation, a bedside chest radiograph was obtained immediately after the patient’s return to the ICU.

Whenever abnormalities in device function were detected, additional chest radiographs were obtained to assess IABP positioning. Minor positional changes were corrected at the bedside, whereas more complex adjustments or significant malposition were evaluated in the cardiac catheterization laboratory under fluoroscopic guidance.

After an initial observation period, patients who were hemodynamically stable, free of active infection, and showed no evidence of recent clinical deterioration were encouraged to perform out-of-bed activities in the standing position under the supervision of the physical therapy team (Figure 3).

**Figure 3:** – Patient receiving axillary intra-aortic balloon pump support during a cardiopulmonary rehabilitation session under the supervision of the multidisciplinary care team. Source: Archives of the Cardiac Catheterization Laboratory, Instituto do Coração, Hospital das Clínicas, Faculdade de Medicina, Universidade de São Paulo.

### Device removal

Device removal was performed at the time determined by the treating team according to each patient’s clinical course. The procedure followed the institutional protocol for femoral introducer removal, avoiding removal during the pharmacological effect of anticoagulants or in the presence of uncontrolled hypertension.

After cessation of IABP counterpulsation, the catheter was removed together with the introducer sheath, when applicable, while maintaining the upper limb in abduction. Hematomas or active bleeding at the puncture site were promptly controlled by adjusting the compression site.

### Statistical analysis

Descriptive statistical analyses were performed. Categorical variables are presented as absolute and relative frequencies. Continuous variables are reported as measures of central tendency and dispersion according to their distribution, which was assessed using the Shapiro-Wilk normality test. Normally distributed variables are expressed as mean ± SD, whereas non-normally distributed variables are presented as median (IQR).

Statistical analyses were performed using RStudio for Windows (version 2022.12.0+353.pro20, “Elsbeth Geranium”). A p-value < 0.05 was considered statistically significant.

### Ethical considerations

The study was approved by the institutional human research ethics committee under CAAE No. 74190123.2.0000.0068, in accordance with Brazilian National Health Council Resolution No. 466/2012, which governs research involving human participants in Brazil. All patients provided written informed consent before undergoing the procedure. Data were handled confidentially in accordance with the Brazilian General Data Protection Law.

## Results

Between December 2020 and December 2023, 22 consecutive patients underwent axillary IABP implantation at a Brazilian referral center for advanced HF.

The clinical and demographic characteristics of the study population are presented in Table 1. The mean age was 53 ± 10.7 years (range, 27–75 years), and 18 patients were male. The most common etiologies of myocardial dysfunction were idiopathic dilated cardiomyopathy (45.5%), Chagas cardiomyopathy (27.3%), and ischemic cardiomyopathy (13.6%). All patients had advanced HF. Nearly all patients were receiving dobutamine (21 patients), including three who were treated with combination therapy with milrinone. A total of 10 patients (45.5%) were receiving intravenous or oral vasodilators, and five (22.7%) were receiving norepinephrine at the time of the procedure. The mean left ventricular ejection fraction (LVEF) was 24.9% ± 7.6%.

**Table 1:** – Baseline characteristics of the study population

Variable	Result (n = 22)
Age, years	53 ± 10.7
Male sex, n (%)	18 (81.8)
NYHA functional class III or IV, n (%)	22 (100)
HF etiology, n (%)	
Idiopathic dilated cardiomyopathy	10 (45.5)
Chagas cardiomyopathy	6 (27.3)
Ischemic cardiomyopathy	3 (13.6)
Other	3 (13.6)
Inotropic therapy, n (%)	12 (95.5)
BMI (kg/m2)	23.0 ± 3.9
LVEF (%)	24.9 ± 7.6
Moderate-to-severe MR, n (%)	12 (54.5)
Hypertension, n (%)	11 (50.0)
DM, n (%)	7 (31.8)
*Creatinine clearance (mL/min)*	60 (IQR: 56-87)
PAD, n (%)	2 (9.1)

BMI: body mass index; DM: diabetes mellitus; HF: heart failure; IQR: interquartile range; LVEF: left ventricular ejection fraction; MR: mitral regurgitation; NYHA: New York Heart Association; PAD: peripheral arterial disease.

The procedure was successfully performed as planned via the left axillary artery in all cases, with no intraprocedural complications, resulting in a technical success rate of 100%. The mean total procedural time was 62.8 ± 21.2 minutes. Patients remained on IABP support for a median of 15 days (IQR, 7-28 days), with a range of 1-65 days (Table 2). During follow-up, 11 patients (50%) experienced favorable clinical outcomes, defined as either successful heart transplantation or hemodynamic stabilization followed by progressive weaning from mechanical circulatory support (Central Illustration).

**Table 2:** – Procedural characteristics and patient outcomes in consecutive order of enrollment

Patient	Age, years	Number of prior IABPs	Length of hospital stay before the procedure, days	Procedure duration, min	Duration of axillary IABP support, days	Complications
1	58	1	45	74	9	Axillary artery pseudoaneurysm (1.5 × 1.0 cm)
2	58	1	58	48	13	Left upper limb ischemia
3	59	0	86	67	26	Device malposition requiring IABP replacement
4	68	1	5	92	7	—
5	64	2	74	58	3	Device malposition not requiring IABP replacement
6	51	2	155	40	29	—
7	52	1	69	88	13	Infection requiring intravenous antibiotic therapy
8	57	1	53	50	35	Stroke
9	75	1	21	77	1	—
10	40	2	93	54	18	—
11	44	1	100	83	65	—
12	45	2	73	50	31	—
13	47	2	151	40	10	—
14	54	1	42	39	9	Device malposition not requiring IABP replacement
15	51	1	124	23	5	Device malposition requiring IABP replacement
16	27	1	79	64	7	—
17	56	1	115	54	55	—
18	55	0	102	49	19	—
19	47	1	177	68	17	—
20	52	1	137	60	38	Left upper limb ischemia
21	67	1	66	108	17	—
22	39	1	71	95	7	—

IABP: intra-aortic balloon pump.

During the period of mechanical circulatory support, nine adverse events were recorded. Two patients (9.1%) developed signs of left upper limb (LUL), one (4.5%) developed bacteremia requiring antibiotic therapy, one (4.5%) experienced a stroke during device removal, one (4.5%) developed an axillary artery pseudoaneurysm, and four (18.2%) experienced device migration (Table 3).

**Table 3:** – Adverse events observed during axillary intra-aortic balloon pump support

Adverse event	n (%)
Device malposition/migration	4 (18.2)
Left upper limb ischemia	2 (9.1)
Bacteremia requiring intravenous antibiotic therapy	1 (4.5)
Stroke	1 (4.5)
Axillary artery pseudoaneurysm	1 (4.5)

## Discussion

The present study reports the initial experience with percutaneous axillary IABP implantation at a Brazilian referral center for advanced HF. The main findings were as follows: i) the percutaneous axillary implantation technique proved to be reproducible, with a high technical success rate; ii) the transaxillary approach enabled early patient mobilization; and iii) the procedure demonstrated a favorable safety profile, with a low complication rate even during the initial implementation phase at our institution.

The first case series describing percutaneous axillary IABP implantation, published in 2013, included approximately 50 patients referred for heart transplantation evaluation.^5^ In that cohort, the mean duration of support was 18 days, and 84% of patients ultimately underwent heart transplantation. Complications requiring intervention included one episode of major bleeding and two cases of LUE ischemia requiring surgical treatment. Device repositioning was necessary in 44% of patients, whereas 20% experienced device malfunction requiring replacement. No deaths, strokes, or device-related infections were reported.

In our cohort, the rate of device migration or malfunction was 18.2% (n = 4), which is lower than the rates reported in the literature (36%-44%).^5,6,10^ One possible explanation is that currently available devices were not specifically designed for axillary access, making adaptation to the anatomy of the subclavian artery and aortic arch more challenging. Devices specifically developed for axillary implantation, such as the NuPulse IVAS (NuPulseCV, North Carolina), have shown promising results for long-term mechanical circulatory support and may reduce the incidence of device malfunction and migration.^11^

Despite these events, all cases had a benign clinical course, and most were resolved with minor bedside positional adjustments without compromising device function. Only two patients experienced migration of the IABP into the axillary artery, preventing complete balloon inflation and requiring fluoroscopy-guided repositioning through a new implantation site. In both cases, no vascular complications occurred, and the patients were subsequently successfully weaned from mechanical circulatory support.

Two patients (9.1%) developed LUL ischemia, characterized by diminished radial and ulnar pulses. In one case, Doppler ultrasonography demonstrated partial thrombosis of the left axillary artery. In both patients, the device was removed, full-dose anticoagulation was initiated, and passive warming of the affected limb was instituted, resulting in restoration of perfusion and complete clinical recovery. No patient required vascular surgical intervention.

Limb ischemia is a well-recognized complication following IABP implantation through either the axillary or femoral approach, with a comparable incidence between the two access sites. The largest published series of patients undergoing axillary IABP implantation reported a limb ischemia rate of 5.3%.^10^ Likewise, a review including 20 studies of transfemoral IABP implantation found a mean limb ischemia incidence of 8.03% (± 7.40%).^12^

In most cases, device removal combined with passive warming of the affected limb is sufficient to reverse this complication. Evidence derived predominantly from studies of transfemoral IABP implantation indicates that limb amputation is uncommon, with a reported incidence ranging from 0.1% to 3.5%.^12^ The use of larger introducer sheaths, female sex, failure to use the sheathless technique, and prolonged duration of device support are among the main risk factors associated with limb ischemia.^12-16^ Boudolas et al. demonstrated that the incidence of this complication increases substantially when device support exceeds 2 days.^13^ Notably, in our series, both cases of limb ischemia occurred in female patients who had prolonged support durations (13 and 38 days).

One patient in the present series experienced a stroke during device removal, identified after the sudden onset of disproportionate right-sided motor weakness. The patient promptly underwent intravenous thrombolysis followed by mechanical thrombectomy, achieving complete neurological recovery. During the same hospitalization, the patient successfully underwent heart transplantation and was discharged without residual neurological deficits.

Prior research indicates that axillary IABP implantation may be associated with neurovascular complications, particularly involving the brachial plexus, presenting as neuropathic pain, paresthesia, or transient motor deficits. These complications are attributed to the close anatomical relationship between the axillary artery and adjacent neural structures, as well as the potential for local compression, hematoma formation, or device malposition. Clinical series and review articles suggest that such events are relatively uncommon but should be considered during procedural planning and execution.^8^ In our cohort, although systematic neurological assessment was not performed, no clinical reports of neuropathic pain or neurological deficits suggestive of brachial plexus injury were documented during follow-up. This finding is consistent with previous studies demonstrating a low incidence of neurological complications when axillary implantation is performed after appropriate anatomical assessment and with meticulous technique.^10^

Boudoulas et al. published a retrospective analysis of 195 patients who underwent percutaneous axillary IABP implantation.^10^ With a mean support duration of 19 days, therapeutic success was achieved in 68.2% of patients, a result comparable to that reported for transfemoral IABP implantation. Only 11% of patients required device removal because of complications.

In our cohort, 50% of patients experienced favorable clinical outcomes, defined as either successful heart transplantation or successful weaning from mechanical circulatory support. This result should be interpreted in light of the severe clinical condition of the study population, characterized by a mean LVEF of 25% (± 7.6%), inotropic support in nearly all patients at the time of implantation, multiple previous invasive procedures, and prolonged hospitalization before the procedure (median, 76.5 days).

Finally, preserving patient mobility deserves particular attention. Among candidates for solid organ transplantation, poorer functional capacity has been associated with increased perioperative complications and prolonged postoperative recovery, adversely affecting post-transplant outcomes.^17^ Furthermore, ambulatory capacity in patients hospitalized with HF has been associated with shorter hospital stays and fewer hospital readmissions.^18^ Therefore, strategies that preserve mobility and functional capacity in patients with advanced HF awaiting heart transplantation should be encouraged. Figure 3 displays the ambulation and physical rehabilitation of a patient receiving IABP support in the ICU.

Taken together, the findings of this case series suggest that percutaneous axillary IABP implantation is a feasible alternative for patients with advanced HF. Strategies aimed at preserving mobility and functional capacity, such as the axillary approach, may represent an important advance in the management of patients awaiting heart transplantation.

### Study limitations

The main limitations of this study include its single-center design, the relatively small sample size, and the lack of direct comparison with other vascular access routes. Multicenter studies involving larger patient populations and additional Brazilian centers with expertise in advanced HF management may help determine whether this technique can be routinely incorporated into clinical practice in our setting.

## Conclusions

This study describes the initial experience with implementing the technique of percutaneous axillary IABP implantation at a Brazilian tertiary cardiology referral center. The clinical outcomes observed were consistent with those reported in the international literature and support the feasibility, effectiveness, and safety of this approach. Nevertheless, further studies are required to define the role of this intervention within the continuum of care for patients hospitalized with advanced HF while awaiting heart transplantation.
